# Tissue-Specific GHR Knockout Mice: Metabolic Phenotypes

**DOI:** 10.3389/fendo.2014.00243

**Published:** 2015-01-19

**Authors:** Liou Y. Sun, Andrzej Bartke

**Affiliations:** ^1^Department of Internal Medicine, Southern Illinois University School of Medicine, Springfield, IL, USA

**Keywords:** GH signaling, mice, GHRKO, hepatic GHR signaling, adipocytes, macrophages

## Abstract

In addition to its major role in the regulation of somatic growth, growth hormone (GH) signaling has profound effects on function of various tissues in the body. However, the cellular location where the GH signaling exerts its effect on metabolic homeostasis remains largely unknown. Here, we briefly review recent progress and insights from mice with GH receptor deletion specifically in adipocytes, macrophages, hepatocytes, pancreatic β-cells, and skeletal muscle cell types. These studies have greatly enhanced our understanding of the GH–IGF-I physiological function.

## Introduction

In addition to its major role in the regulation of somatic growth (1), growth hormone (GH) is a major regulator of gene expression and protein synthesis in the liver, fat mobilization in the metabolically active tissues, and organ response to insulin (1). Hepatocytes, pancreatic beta cells, adipocytes, macrophages, and muscle cells are among the cell types that express GH receptors and are critical targets for GH action. Effects of GH on these cells are thought to be predominantly direct, whereas many of its effects are mediated through circulating insulin-like growth factor I (IGF-I) or local (auto/paracrine) IGF-I actions. In 1997, the Kopchick laboratory reported creating mice with targeted deletion (“knockout”) of the GH receptor and they resulting profound GH resistance, reduction of circulating IGF-I levels, disruption of the negative IGF-I feedback, and increase in plasma levels of GH (2). Studies of these animals provided much new information about physiological actions in mammals (3). Mice homozygous for GHR deletion (GHR-KO mice) have markedly increased adiposity and yet are metabolically “healthy” with reduced levels of insulin, reduced or normal levels of glucose, enhanced insulin sensitivity, increased adiponectin levels, reduced blood pressure, and significantly extended longevity of both females and males (3). The fascinating consequences of the global deletion of GH receptors in GHR-KO mice stimulated interest in identifying the role of GH signaling in different target organs of this hormone. It is believed that GH exerts systemic effects primarily through liver-derived IGF-I and adipocyte-derived circulating proteins, such as adipokines and cytokines. Surprisingly, liver-specific IGF-I deletion in mice (LID) (4) has almost no effect on postnatal somatic growth. Moreover, LID mice were found to have hyperinsulinemia and muscle insulin resistance (5). Insulin resistance of these animals was ascribed to stimulation of GH release in response to reduced levels of IGF-I in the circulation. In support of this interpretation suppressing GH level by GH-releasing hormone antagonist dramatically increased insulin sensitivity in these mice (5). These data suggest that GH plays a direct role in systemic carbohydrate metabolism, glucose homeostasis, and insulin action. However, the cellular location of the GH actions remains largely unknown. In this article, we will briefly review the results of studies aimed at identifying the role of different organs or cell types in mediating the physiological effects of GH.

## Tissue-Specific GHR Signaling

**Figure 1:**
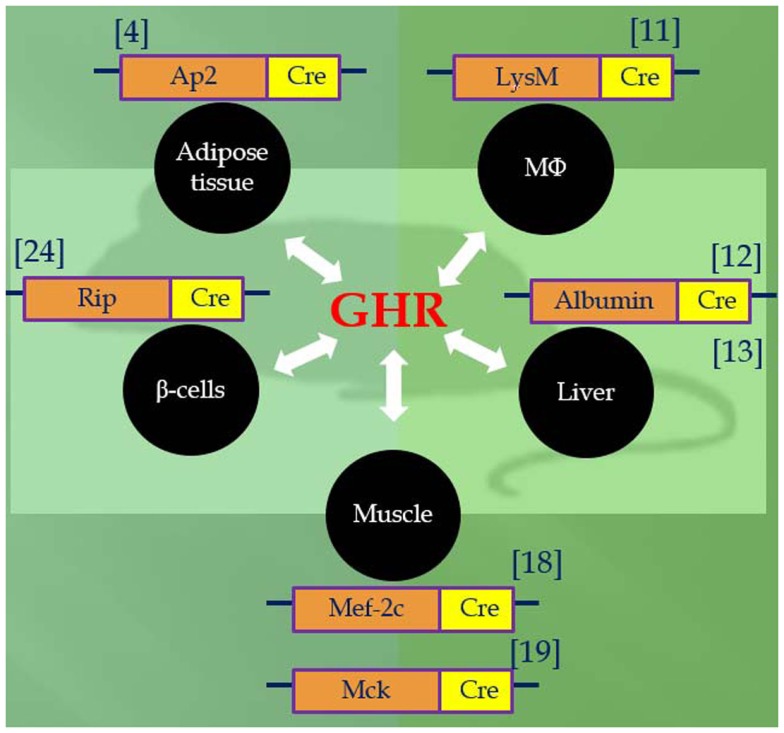
**Tissue-specific GHR knockouts using promoter Cre-loxP recombination**.

Utilizing the Cre/LoxP system, recent studies of mice with GH receptor deletion specifically in various cell types have greatly enhanced our understanding of the GH–IGF-I axis. Results obtained in these animals provide novel insights into the critical role of the local GH signaling in the particular tissues. Published evidence indicated that each of these organ-specific GHRKO mice has its own unique set of physiological and metabolic features. Some of the findings are consistent with the results obtained in the whole-body GHR null mice, while others are seen only in specific organ GHRKO mutants, suggesting that the existence of previously unknown interactions between GH target tissues. The most important phenotypical changes in mice with organ-specific disruption of GHR signaling are summarized in Table 1 and Figure 1 and are discussed below.

**Table 1:** **Major phenotypical and metabolic changes in mice with organ-specific disruption of GHR signaling**.

Name	Tissue	Cre	Life Span	Strain	Obesity	Blood GH	Blood IGF-I	Insulin sensitivity	Glucose tolerance	Other metabolic changes
GHRKO	Whole-body		↑	Ola-BALB/c	↑	↑	↓	↑	↑	↑ Adiponectin, leptin
Fat-GHRKO	Adipocytes	aP2	?	C57BL/G	↑	N.C.	N.C.	N.C.	N.C.	
Mac-GHRKO	Macrophages	LysM	?	C57BL/6	↑HFD		N.C.	↓HFD	↓HFD	Metabolic normal fed with normal chow
Liv-GHRKO	Hepatocytes	Albumin	?	129/SvJ-C57BL/6	N.C.	↑	↓	↓	↓	Impaired lipid homeostasis and hepatic steatosis (6)
Liv-GHRKO	Hepatocytes	Albumin	?	C57BL/6	N.C.	↑	↓	N.C.	N.C.	Fatty liver phenotype only in male (7)
β-GHRKO	Pancreatic β-cells	Rip	?	C57BL/6	N.C.		N.C.	↓HFD	↓HFD	Lost the first–phase GSIS and glucose intolerance on HFD
Mus-GHRKO	Muscle cells	Mef-2c	?	129/SvJ-C57BL/6	↑	?	?	↓	↓	Marked metabolic abnormalities (8)
Mus-GHRKO	Muscle cells	Mck	?	C57BL/6 × C3H	↓	N.C.	N.C.	↑	↑	Decreased systemic inflammation, muscle and hepatic triglyceride content (9)

*?, Information not available*.

## GHR Signaling Disruption in Adipocytes and Macrophages

Growth hormone exerts profound effects on adipose tissue. Overall, GH regulates adipocyte proliferation and differentiation and decreases fat deposition by both inhibiting triglyceride accumulation and increasing lipolysis. Furthermore, GH has been shown to negatively regulate the secretion of adipokines such as leptin and adiponectin. Compared to littermate control mice, the whole-body GHR null mice have higher percent body fat with elevated circulating adiponectin and leptin levels. Intriguingly, these mutant mice are found to have preferential accumulation of subcutaneous fat depots, while visceral fat pads in animals with different genetic background are either less affected or diminished in size as compared to the control mice.

List et al. have recently disrupted GHR in white adipose tissue (WAT) under the aP2-cre promoter/enhancer and produced Fat-GHRKO mice (10). Fat-GHRKO mice share some characteristics with whole-body GHRKO mice; they are obese with increased total body fat, enlarged adipocyte size, and increased circulating leptin levels (10). However, unlike global GHR null mice, these mice show no signs of metabolic improvements and have normal glucose homeostasis and normal/decreased levels of adiponectin (10). In interpreting these unexpected findings, it is necessary to consider that GHR was likely deleted not only in adipocyte but also in other cell types, including macrophages (11). Further studies using different Cre lines of mice, such as adiponectin-Cre transgenic mice, would be necessary to elucidate the direct role of GH in adipocytes from different depots. We would also add that much was learned about the role of insulin signaling in adipose tissue from studies in FIRKO mice, which were generated using the same aP2-cre Tg mice (12).

Infiltration into adipose tissue by macrophage (MΦ) has emerged as an important component in the pathogenesis of diet-induced obesity (DIO) and insulin resistance (13). The invading macrophages have been shown to be a major source of proinflammatory cytokines, such as IL-1β and TNF-α (14), and thus, are contributors to the establishment of the low-grade chronic inflammatory state associated with obesity. In order to understand the role of GH signaling in macrophage invasion, MΦ-specific GH receptor-KO (MacGHR-KO) mice were created in R. K. Menon’s group by crossing LysMcre mice with GHR exon 4 floxed mice (15). Fed with a normal chow diet, MacGHR-KO exhibited normal growth profiles and metabolic characteristics (15). Compared to normal animals fed HFD, MacGHR-KO mice on the same diet had greater impairments of glucose tolerance and insulin sensitivity as revealed by insulin and glucose tolerance tests (15). Moreover, increased visceral WAT, enlarged adipocyte size, increased MΦ invasion, elevated proinflammatory cytokines, and attenuated insulin-stimulated Akt phosphorylation were detected in the WAT of HFD fed MacGHR-KO mice. These results suggest that MΦ-specific GH signaling plays an important role in the pathogenesis of DIO.

## Hepatic GHR Signaling

Hepatic GHRs have been shown to be expressed at high levels and therefore the liver is highly responsive to GH signaling. The liver is also the major site of IGF-I secretion and the main source of circulating IGF-I. Due to absence of negative feedback of serum IGF-I, LID mice have high circulating level of GH. The elevated GH secretion counteracts insulin action, decreasing insulin sensitivity, and glucose tolerance (5). These results are consistent with the ability of GH to affect various tissues via direct action, which might be independent of IGF-I. However, the precise contribution of GH action liver remains to be elucidated.

Using a similar approach, two independent groups have generated two lines of mice with liver-specific disruption of GHR (LivGHR-KO) under the albumin-cre driver (6, 7). Fan et al. reported that LivGHR-KO mice have normal body size and bone linear growth despite profound suppression of circulating IGF-I level (6). Subsequently, List et al. found that Liv-GHRKO mice generated in Kopchick’s group were smaller than littermate controls, with their body length and body weight significantly decreased in both sexes (7). In the first report (6), LivGHR-KO mice showed the characteristics of metabolic abnormalities including hyperinsulinemia, impaired insulin signaling, abnormal glucose intolerance, hepatic steatosis, and altered lipid homeostasis. In contrast, in the second line of LivGHR-KO mice (7), all these metabolic parameters remained unaltered as compared to the controls except that fatty livers were manifested in male LivGHR-KO mice. The difference in genetic backgrounds and the diet employed in these two studies may account for the different phenotypes.

## GHR Signaling in Skeletal Muscle

Growth hormone–IGF axis has a dramatic impact on skeletal muscle growth and anabolism. GH has been shown to stimulate cell mitosis, increase protein translation, promote satellite cell proliferation, and inhibit apoptosis (16, 17). There is also evidence that GH can affect muscle fiber composition (18). However, it will be important to distinguish between direct actions of GH and secondary IGF-I mediated GH effects. Using whole-body GHRKO mice, Sotiropoulos et al. found that GH enlarged skeletal muscle size by increasing myofiber size in the IGF-I independent manner (19). Interestingly, they have shown that GH had no impact on cell number, proliferation, or differentiation of the progenitor cells (19). These data indicate the importance of GH in skeletal muscle physiology via IGF-I independent mechanisms responsible for these effects.

The Clemens laboratory has recently characterized the novel line of skeletal muscle-specific GHR (MusGHR-KO) and IGF-IR (MusIGFR-KO-1) knockout mice under myocyte-specific enhancer factor 2C (mef-2c-73k) promoter (8). In contrast with MusIGFR-KO mutant mice, MusGHR-KO-1 mice exhibited marked metabolic abnormalities including peripheral obesity, insulin resistance, and impaired glucose tolerance. Intriguingly, a second line of skeletal muscle-specific GHR knockout (MusGHR-KO-2) mice developed by LeRoith’s group [using muscle creatine kinase (MCK) promoter] were found to have the opposite metabolic phenotype (9). The MusGHR-KO-2 mice had reduced fat mass, improved metabolic markers when fed with normal diet, and were protected against metabolic impairments in the high-fat-diet settings. Taken together, these studies have indicated that that GHR signaling in different subsets of muscle cells could have different metabolic effects. There remain important areas for further investigation. Furthermore, in interpreting these findings using mef-2c and MCK cre transgenic mice, it is necessary to consider that GHR was likely deleted not only in skeletal muscle cells but also in cardiac muscles.

## GHR Signaling Disruption in Beta cells

The ability of pancreatic β-cells to synthesize and secrete insulin is the key to overcome the hyperglycemia and maintain the glucose homeostasis in the settings of insulin resistance. Impairment to secreting sufficient insulin levels in the face of hyperglycemia leads to the pathogenesis of diabetes (20). GH has been shown to play an important role in maintaining pancreatic β-cells mass (21), regulating β-cell proliferation (22), and differentiation (23) and stimulating gene expression of components in the insulin signaling pathway in these cells.

In order to elucidate the specific role of GH signaling pancreatic β-cells. Wu et al. generated β cell-specific GHR null mice (βGHRKO) employing RIP-Cre recombinase (24). βGHRKO mice developed impaired glucose-stimulated insulin secretion (GSIS) while no alteration was revealed in GTT, ITT test, or β cell staining. Intriguingly, when fed with HFD, βGHRKO mice lost the first-phase GSIS and developed exacerbated glucose intolerance. These mutant mice exhibited a dramatic defect in β cell hyperplasia due to decreased cell proliferation in response to HFD challenge. These data point out that GH signaling in β cells is pivotal in the process of compensatory cell proliferation and insulin secretion in the state of the DIO.

## Perspectives

There has been considerable recent progress in unraveling the effects and mechanisms of tissue-specific actions of GH signaling. However, many gaps remain in our understanding of these processes. For example, one future imperative will be to explore the GH action and signaling in tissues for which GH action has not been clearly evaluated. In particular, further study of key cell populations at the specific brain regions or in certain brain cell populations will be critical, with special attention to (1) hypothalamus, which is considered a key regulator of food intake and energy expenditure in mammals; (2) hippocampus, which is critical for learning and memory and is sensitive to hormonal fluctuations; and (3) glial cells including astrocytes, which have emerged as active players in CNS metabolic activities. It will be important to use these novel tissue/cell-specific GHR null mice to further dissect the functioning of the somatotropic axis, elucidate the mechanisms of cross-talk among different tissues, and determine the interactions between genes and environment in the context of development and aging. Such studies will offer critical insight into the basic physiological processes and molecular mechanisms that regulate metabolism and insulin action.

## Conflict of Interest Statement

The authors declare that the research was conducted in the absence of any commercial or financial relationships that could be construed as a potential conflict of interest.
